# Application of Group Method of Data Handling on the Ultimate Conditions’ Prediction of FRP-Confined Concrete Cylinders

**DOI:** 10.3390/polym14173615

**Published:** 2022-09-01

**Authors:** Chubing Deng, Ruiliang Zhang, Xinhua Xue

**Affiliations:** State Key Laboratory of Hydraulics and Mountain River Engineering, College of Water Resource and Hydropower, Sichuan University, Chengdu 610065, China

**Keywords:** FRP-confined concrete cylinder, fiber-reinforced polymer (FRP), group method of data handling (GMDH), ultimate conditions, machine learning

## Abstract

Fiber-reinforced polymer (FRP) is widely used in the field of structural engineering, for example, as a confining material for concrete. The ultimate conditions (i.e., compressive strength and ultimate axial strain) are key factors that need to be considered in the practical applications of FRP-confined concrete cylinders. However, the prediction accuracy of existing confinement models is low and cannot provide an effective reference for practical applications. In this paper, a database containing experimental data of 221 FRP-confined normal concrete cylinder specimens was collected from the available literature, and eleven parameters such as the confining stress, stiffness ratio and strain ratio were selected as the input parameters. Then, a promising machine learning algorithm, i.e., group method of data handling (GMDH), was applied to establish a confinement model. The GMDH model was compared with nine existing models, and the prediction results of these models were evaluated by five comprehensive indicators. The results indicated that the GMDH model had higher prediction accuracy and better stability than existing confinement models, with determination coefficients of 0.97 (compressive strength) and 0.91 (ultimate axial strain). Finally, a convenient graphical user interface (GUI) was developed, which can provide a quick and efficient reference for engineering design and is freely available.

## 1. Introduction

Fiber-reinforced polymer (FRP) has the advantages of fatigue resistance, high corrosion resistance, high strength, light weight, convenient construction and good reinforcement effect, and is widely used in the field of structural engineering. For example, as a confining material for concrete [1,2,3]. Currently, the concrete types of FRP-confined concrete include normal-strength concrete (NSC), high-strength concrete (HSC), reinforced concrete (RC), recycled aggregate concrete (RAC) and ultra-high-performance concrete (UHPC). There are many kinds of FRP composites, mainly AFRP (aramid), CFRP (carbon) and GFRP (glass).

Numerous studies have shown that the strength and ductility of concrete members can be significantly improved by applying lateral restraints through FRP jackets [4,5,6]. From an engineering point of view, the improvement of the bearing capacity and ductility of the specimen after being confined is the most concerned. Therefore, in the design process of FRP-confined concrete, the high-precision prediction of compressive strength and ultimate axial strain can provide an important reference for the determination of parameters such as the number of wrapping layers and the nominal thickness of the FRP jacket. Many studies have been conducted [6,7,8,9,10,11,12,13,14,15]. Benzaid et al. [6] conducted, respectively, uniaxial compression tests on normal- and reinforced-concrete cylinders strengthened with external CFRP to study their axial and transverse mechanical properties. By analyzing the test data, a simple confinement model of FRP-confined columns was proposed. Teng et al. [9] analyzed the stress–strain model proposed by Spoelstra and Monti [13] and the experimental data of 48 FRP-confined concrete cylinder specimens, and proposed more accurate expressions for the compressive strength and ultimate axial strain. These expressions allowed the influence of FRP strain capacity and stiffness ratio to be reflected separately. Khorramian et al. [15] established a new mechanics-based confinement model based on 788 sets of experimental data.

Considering that experimental research requires a lot of manpower and machine equipment, in recent years, many scholars have applied some machine learning algorithms to the field of engineering [16,17,18,19,20,21]. Ruggieri et al. [17] proposed a machine learning framework for the vulnerability analysis of existing buildings, which opens up a new scenario in the field of risk mitigation strategies and vulnerability assessment procedures. Keshtegar et al. [19] successfully applied the chaos control method to the ultimate strength and strain prediction of FRP-confined concrete cylinders and proposed three nonlinear confinement models. Jawdhari et al. [20] used multiple nonlinear regression (MNR) method to establish a confinement model including the damage effects. Sadeghian and Fam [21] established a confinement model based on regression analyses on experimental data of 518 cylindrical concrete specimens. Mansouri et al. [22] studied the ultimate conditions of FRP-confined concrete cylinders (including NSC, RC and UHPC) through adaptive neural fuzzy inference system with subtractive clustering (ANFIS-SC), radial basis neural network (RBNN) and M5 model tree (M5 Tree). In addition, Cevik et al. [23,24,25] studied the application of soft computing methods such as multiple adaptive regression spline (MARS) and genetic programming (GP) in the prediction of ultimate conditions of FRP-confined concrete cylinders, and found that soft computing methods can make important contributions to the prediction of ultimate conditions.

At present, most research studies on FRP-confined concrete mainly focus on the prediction of compressive strength, and the research on predicting the ultimate axial strain is very limited. Moreover, the existing confinement models have poor performance in predicting the ultimate axial strain and cannot provide an effective reference for practical applications. Therefore, in order to propose a high-precision confinement model that can provide a more effective reference for engineering design, this paper established a database containing experimental data of 221 FRP-confined normal concrete cylinder specimens [3,8,23,24,25,26,27,28,29,30,31,32,33,34,35,36,37,38,39,40,41,42,43,44,45] and applied a promising machine learning technique (i.e., GMDH), which has been widely used in the field of engineering, to establish a new confinement model.

## 2. Group Method of Data Handling

**Figure 1:**
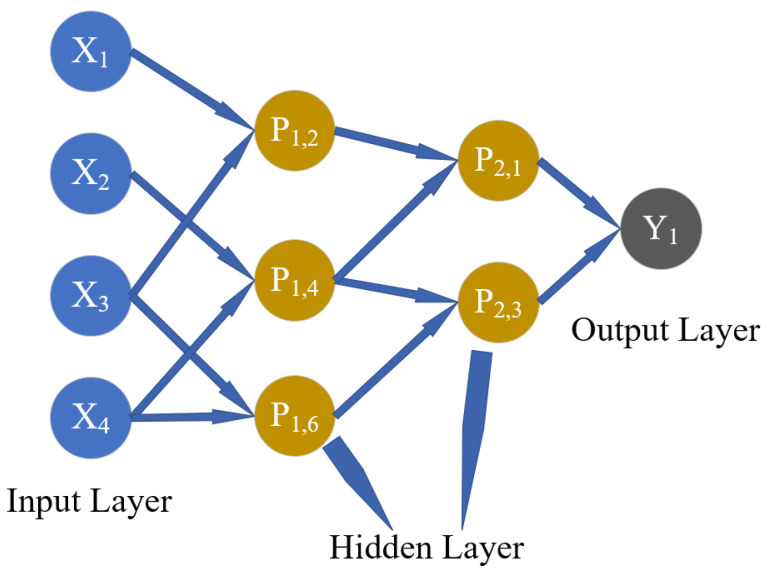
Typical network structure of the GMDH model.

As an inductive learning algorithm, GMDH was created by Ivakhnenko in 1971 [46] and has been widely used in the civil engineering field [7,47,48]. Due to the structure of the feedforward neural network, the GMDH network is also called a polynomial neural network [49]. The GMDH model consisting of one output and four input neurons is shown in Figure 1.

Here, $X_{1}\;\sim \;X_{4}$ are the input neurons (input parameters) of the model, $P_{1,1}\;\sim \;P_{2,3}$ are the hidden neurons of the model and $Y_{1}$ is the output neuron (output parameter) of the model.

**Figure 2:**
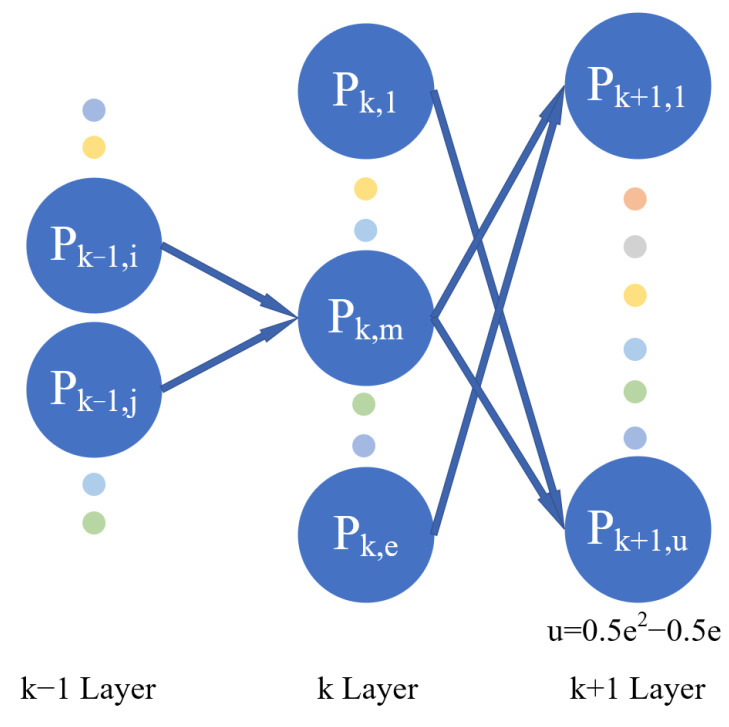
The production of neurons.

GMDH network can be divided into three parts: (1) the input layer, (2) the hidden layer, and (3) the output layer. First, the neurons of the input layer are combined in pairs to generate the neurons of the first layer. Assuming that there are e neurons in a certain layer of GMDH network, the next layer will generate $C_{e}^{2}=0.5e^{2}-0.5e$ neurons (as shown in Figure 2). According to the GMDH network, the model is proposed as a set of neurons. The relationship between neurons in different layers satisfies the Ivakhnenko polynomial relationship, as shown in Equation (1) (taking the neuron $P_{k,m}$ in Figure 2 as an example). Therefore, for a given learning sample, the output values of each neuron in a certain layer are firstly calculated. The least square method is used for polynomial coefficient fitting and thus the output value of each neuron in the next layer is as close to the actual value as possible.

(1) $$P_{k,m}=C_{1}+C_{2}P_{k-1,i}+C_{3}P_{k-1,j}+C_{4}P_{k-1,i}{}^{2}+C_{5}P_{k-1,j}{}^{2}+C_{6}P_{k-1,i}P_{k-1,j}$$

where $P_{k,m}$ represents the output value of the *m*th neuron in layer *k*, and $P_{k-1,i}$ and $P_{k-1,j}$ represent the output values of the *i*th and *j*th neurons in layer *k* − 1, respectively. $P_{0,i}$ represents the input value of the *i*th neuron in the input layer $X_{i}$, and $C_{1}\;\sim \;C_{6}$ are the polynomial coefficients.

**Figure 3:**
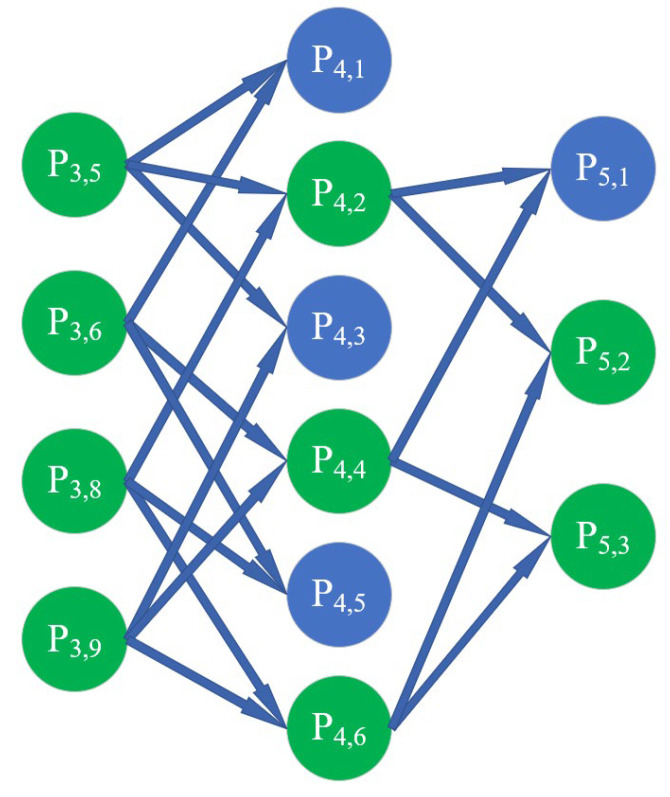
Selection of neurons in the same layer.

In the GMDH network, the selection of neurons is carried out according to the mean square deviation of the output value corresponding to each neuron. All neurons in the same layer are compared one by one with a preset threshold. If the mean square deviation of a neuron is higher than the threshold, the neuron will be eliminated. If not, it will be retained. Among the retained neurons, the part with a smaller error is selected as the basis for generating the neurons of the next layer. Figure 3 is a schematic diagram of the generation of neurons in the next layer. The above steps are repeated until the error of neurons in the next layer is larger than that of the previous layer; then, the neuron with the smallest mean square deviation is taken as the final output of the system. Thus far, the constructed neural network model is the optimal model. The general connections between the output and input parameters can be expressed by the Kolmogorov–Gabor polynomial [49], as shown in Equation (2).

(2) $$\begin{array}{ll} Y_{1} & =f\left(X_{1},X_{2},X_{3},\ldots \right)\\ & =C_{0}+\overset{n}{\underset{i=1}{\sum }}C_{i}X_{i}+\overset{n}{\underset{j=1}{\sum }}\overset{n}{\underset{i=1}{\sum }}C_{ij}X_{i}X_{j}+\overset{n}{\underset{k=1}{\sum }}\overset{n}{\underset{j=1}{\sum }}\overset{n}{\underset{i=1}{\sum }}C_{ijk}X_{i}X_{j}X_{k}+\ldots \end{array}$$

## 3. Database

**Figure 4:**
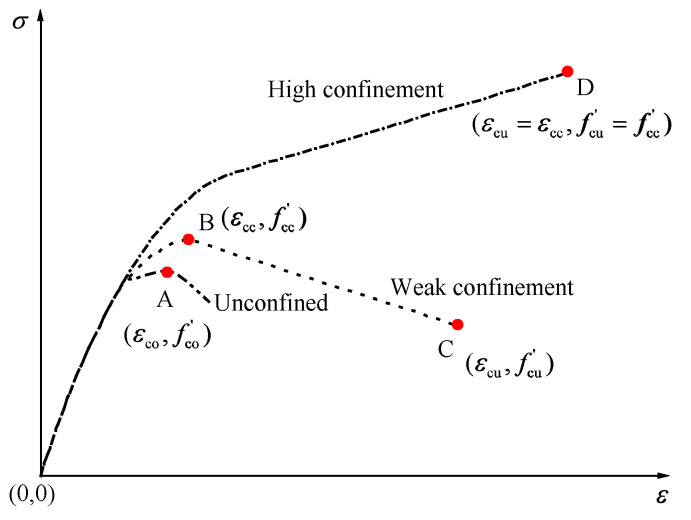
Typical axial stress–strain curves of FRP-confined concrete.

The typical axial stress–strain curves of FRP-confined concrete are shown in Figure 4, and the ultimate conditions in this paper refer to compressive strength $f_{cc}^{\prime }$ and ultimate axial strain.

**Figure 5:**
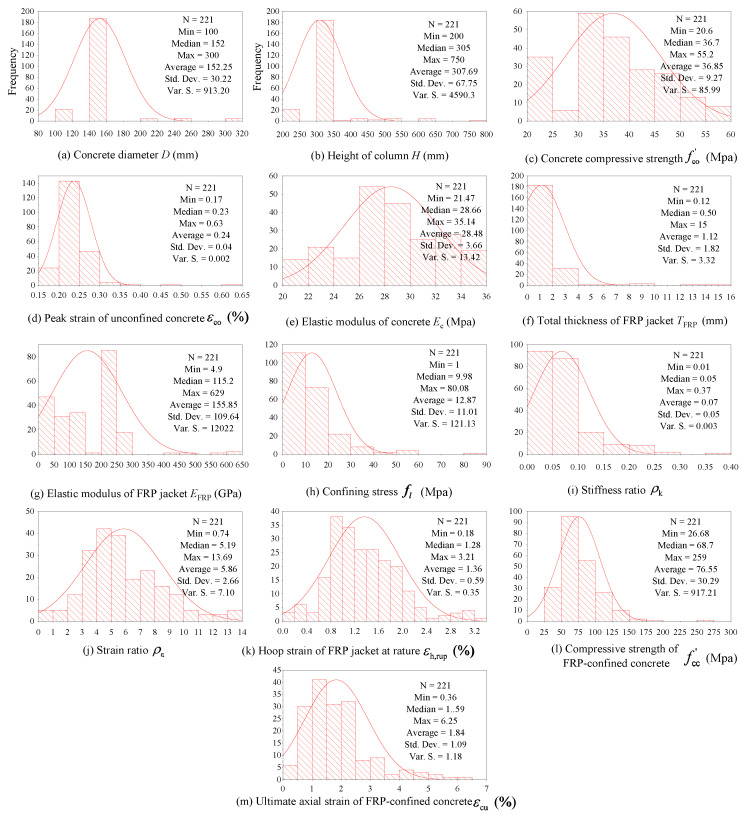
Frequency histogram of the experimental data.

In this paper, a database containing experimental data of 221 normal strength concrete cylinder specimens restrained by various types of FRP composites was collected from the available literature [3,8,23,24,25,26,27,28,29,30,31,32,33,34,35,36,37,38,39,40,41,42,43,44,45]. The database meets the following requirements:

(1) The collected parameters include: the diameter of unconfined concrete D, the height of unconfined concrete H, the elastic modulus of unconfined concrete $E_{c}$, the peak strain of unconfined concrete $\varepsilon_{co}$, the peak strength of unconfined concrete $f_{co}^{\prime }$, the total thickness of the FRP jacket $T_{FRP}$, the elastic modulus of the FRP jacket $E_{FRP}$, the hoop strain of the FRP jacket at rapture $\varepsilon_{h,rup}$, confining stress $f_{l}$, stiffness ratio $\rho_{k}$, strain ratio $\rho_{\varepsilon }$, the compressive strength of FRP-confined concrete $f_{cc}^{\prime }$ and the ultimate axial strain of FRP-confined concrete $\varepsilon_{cu}$ (Note: The units of parameters are shown in Figure 5). If the test values of $E_{c}$ and $\varepsilon_{co}$ were not provided, to make the results more accurate, Equations (3) and (4) were adopted [50,51].

(3) $$E_{c}=4730\sqrt{f_{co}^{\prime }}$$

(4) $$\varepsilon_{co}=9.37\sqrt[{4}]{f_{co}^{\prime }}\times 10^{-4}$$

(2) The height-to-diameter ratio of each specimen is less than 3 to exclude the influence of specimen slenderness.
(3) The type of concrete is normal-strength concrete (NSC).
(4) Only the specimens that failed due to FRP rupture at the ultimate condition are included.
(5) Only the fully FRP-confined specimens are included, and the fiber direction is the hoop direction.

Figure 4 shows the frequency histograms of these parameters, which can visually represent the distributions of the data.

In addition, Pearson correlation analysis was conducted on all data to initially understand the correlation between the ultimate conditions and various parameters, and this can provide a reference for the input parameter selection of the model (as shown in Table 1). The results indicate that the correlations between the ultimate conditions and four parameters (i.e., confining stress $f_{l}$, stiffness ratio $\rho_{k}$, total thickness of the FRP jacket $T_{FRP}$ and concrete compressive strength $f_{co}^{\prime }$) are significant.

## 4. Existing Confinement Models

**Figure 6:**
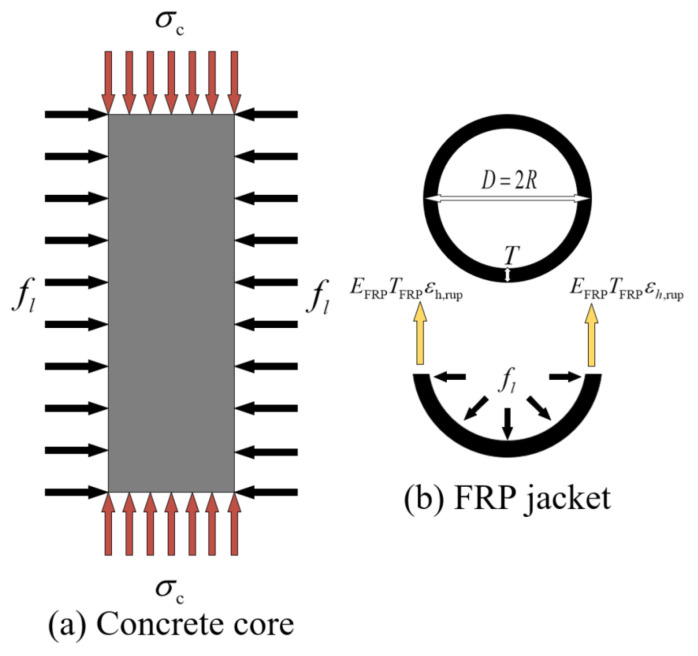
The mechanical analysis of concrete core.

Figure 6 is a schematic diagram of the mechanical analysis of a normal concrete cylinder under the constraint of the FRP composite. In the process of constraining normal concrete cylinder by FRP composite, the FRP composite provides passive-restraint force. Thus, the concrete column will expand around under the action of axial load and the FRP composite passively restricts the deformation of the concrete core.

The interaction force between the two is the lateral confining stress $f_{l}$. Assuming that it is uniformly distributed in the lateral direction, the confining stress can be calculated by Equation (5).

(5) $$f_{l}=\frac{2\sigma_{h}T_{FRP}}{D}=\frac{\sigma_{h}T_{FRP}}{R}$$

where $\sigma_{h}$ represents the hoop tensile stress of the FRP jacket and R and D represent the radius and diameter of unconfined concrete cylinder, respectively. $T_{FRP}$ represents the total thickness of the FRP jacket.

(6) $$T_{FRP}=nt_{FRP}$$

(7) $$\sigma_{h}=\varepsilon_{h,rup}E_{FRP}$$

where *n* represents the number of wrapping layers, $t_{FRP}$ represents the nominal thickness of the FRP jacket, $\varepsilon_{h,rup}$ represents the hoop strain of the FRP jacket at rupture and $E_{FRP}$ represents the elastic modulus of the FRP jacket.

Many scholars have studied the ultimate conditions of cylindrical concrete confined by various types of FRP composites through theoretical analysis, experimental research and neural network methods, and proposed nearly 80 prediction models [4,6,9,10,11,31,52,53,54]. To determine whether the GMDH model has application prospect, it is necessary to compare and analyze it with nine existing models, and nine selected models are listed in Table 2.

## 5. Establishment and Evaluation of the GMDH Model

### 5.1. Comprehensive Indicators

In this paper, five comprehensive indicators, i.e., determination coefficient (R^2^), coefficient of variation (COV), mean absolute error (MAE), root mean square error (RMSE) and mean absolute percentage error (MAPE), are selected to evaluate the prediction results of the model, and the relevant calculation formulas are as follows:

(8) $$R^{2}=\frac{{\left(\sum_{i=1}^{k}\left(\bar{X}-X_{i}\right)\left(\bar{Y}-Y_{i}\right)\right)}^{2}}{\sum_{i=1}^{k}{\left(\bar{X}-X_{i}\right)}^{2}\sum_{i=1}^{k}{\left(\bar{Y}-Y_{i}\right)}^{2}}$$

(9) $$\mathrm{COV}=\frac{\sqrt{\frac{1}{k}\sum_{i=1}^{k}{\left(\frac{Y_{i}}{X_{i}}-\frac{1}{k}\sum_{i=1}^{k}\frac{Y_{i}}{X_{i}}\right)}^{2}}}{\frac{1}{k}\sum_{i=1}^{k}\frac{Y_{i}}{X_{i}}}$$

(10) $$\mathrm{RMSE}=\sqrt{\frac{\sum_{i=1}^{k}{\left(Y_{i}-X_{i}\right)}^{2}}{k}}$$

(11) $$\mathrm{MAE}=\frac{1}{k}\overset{k}{\underset{i=1}{\sum }}\left|Y_{i}-X_{i}\right|$$

(12) $$\mathrm{MAPE}=\frac{1}{k}\overset{k}{\underset{i=1}{\sum }}\left|\frac{Y_{i}-X_{i}}{X_{i}}\right|$$

where $Y_{i}$ and $X_{i}$ represent the predicted and measured values, respectively, *k* represents the total number of FRP-confined normal concrete cylinder specimens and $\bar{Y}$ and $\bar{X}\;$ represent the average values of the predicted and measured values, respectively.

### 5.2. Selection of Input Parameters

When establishing the confinement model of FRP-confined concrete, the input parameters mainly include two types. One is the measured parameters, such as the diameter of concrete core D and hoop strain of the FRP jacket at rapture $\varepsilon_{h,rup}$. The other is the new parameters formed by the combination of measured parameters, such as the confining stress $f_{l}$, stiffness ratio $\rho_{k}$ and strain ratio $\rho_{\varepsilon }$. According to Magao’s research results, different combinations of the input parameters will affect the prediction results of the model [55]. Therefore, this paper considered establishing models with four different input forms to obtain the optimal form, and the relevant input forms are shown in Table 3. The input parameters of model A are all measured parameters (original parameters), and the input parameters of model B are three new parameters. The input parameters of model C are determined by referring to the model proposed by Teng et al. [9] and the results of Pearson correlation analysis, and the input parameters of model D consist of the measured parameters and three new parameters. Obviously, the output parameters of the model are compressive strength $f_{cc}^{\prime }$ and ultimate axial strain $\varepsilon_{cu}$.

### 5.3. Network Configuration

Experimental data are randomly divided into the training set (177 groups) and test set (44 groups) according to the ratio of 2:1. In the GMDH-type neural network, the prediction accuracy of the train set will increase with the increase in the number of hidden layers, and the overfitting phenomenon will occur when the number of hidden layers reaches a certain level. Specifically, the prediction accuracy of the training set is significantly higher than that of the test set. To prevent the occurrence of the overfitting phenomenon, the number of hidden layers and neurons in a single layer are initially set to 5 and 15, and the values of the two parameters are continuously adjusted according to the training results.

### 5.4. The Optimal Input Form

**Figure 7:**
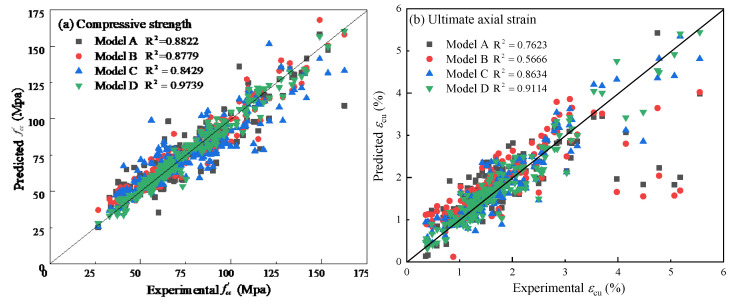
The prediction results of the four models corresponding to four input forms.

**Figure 8:**
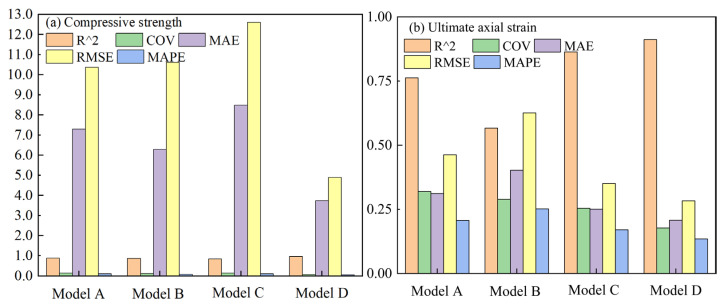
Comparison of the prediction results of the four models.

The prediction results of the four GMDH models corresponding to the four input forms are shown in Figure 7, and the comparison of the prediction results of these models is shown in Figure 8.

As can be seen from Figure 7 and Figure 8, the prediction results of the four GMDH models corresponding to the four input forms agree well with the measured results, indicating that the GMDH-type neural network has good application potential regarding the prediction of the ultimate conditions of FRP-confined concrete. For the compressive strength prediction, model D is superior to models A, B and C. Additionally, its determination coefficient is 0.954, while the determination coefficients of the other three models are 0.899, 0.888 and 0.890, respectively. For the ultimate axial strain prediction, model D is better than the other three models, and its determination coefficient is 0.911, while the determination coefficients of the other three models are 0.762, 0.567 and 0.863, respectively. It can be seen from the above results that the model corresponding to the fourth input form (model D, hereinafter referred to as GMDH model) is better than the models corresponding to the other three input forms, and both original and new data should be taken into account when establishing the ultimate conditions model through the GMDH-type neural network.

**Figure 9:**
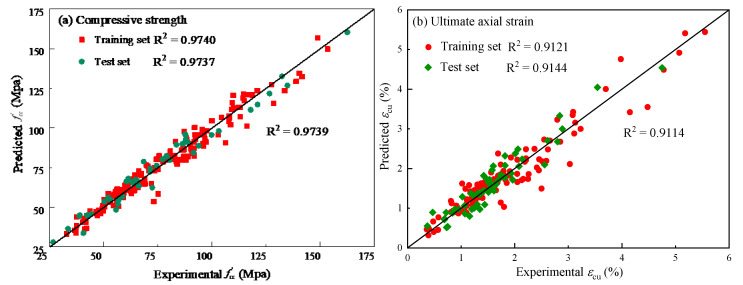
Comparison of the prediction results between the training and test sets.

In the training process of the model, the number of hidden layers should not be too large, otherwise the overfitting phenomenon can easily occur. To determine whether the GMDH model proposed in this paper has an overfitting phenomenon, the prediction results of the training set and test set of the GMDH model are analyzed, as shown in Figure 9. For the prediction of ultimate axial strain of GMDH model, the determination coefficients of the test set are higher than those of the training set. For the compressive strength prediction, the determination coefficient of the training set is higher than that of the test set, but the difference is very small. This shows that the setting of the number of hidden layers is reasonable.

### 5.5. Comparison with Existing Models

**Figure 10:**
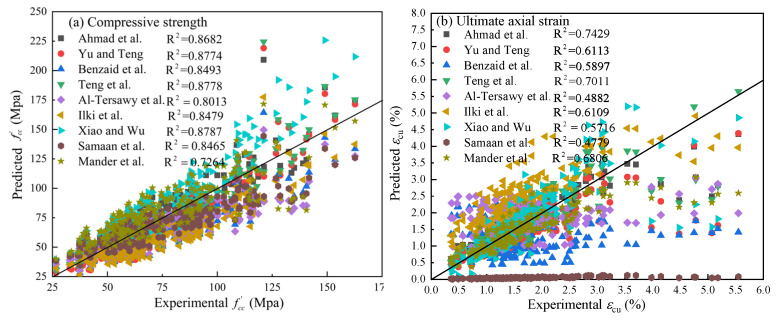
Prediction results of nine existing models [4,6,9,10,11,31,52,53,54].

**Figure 11:**
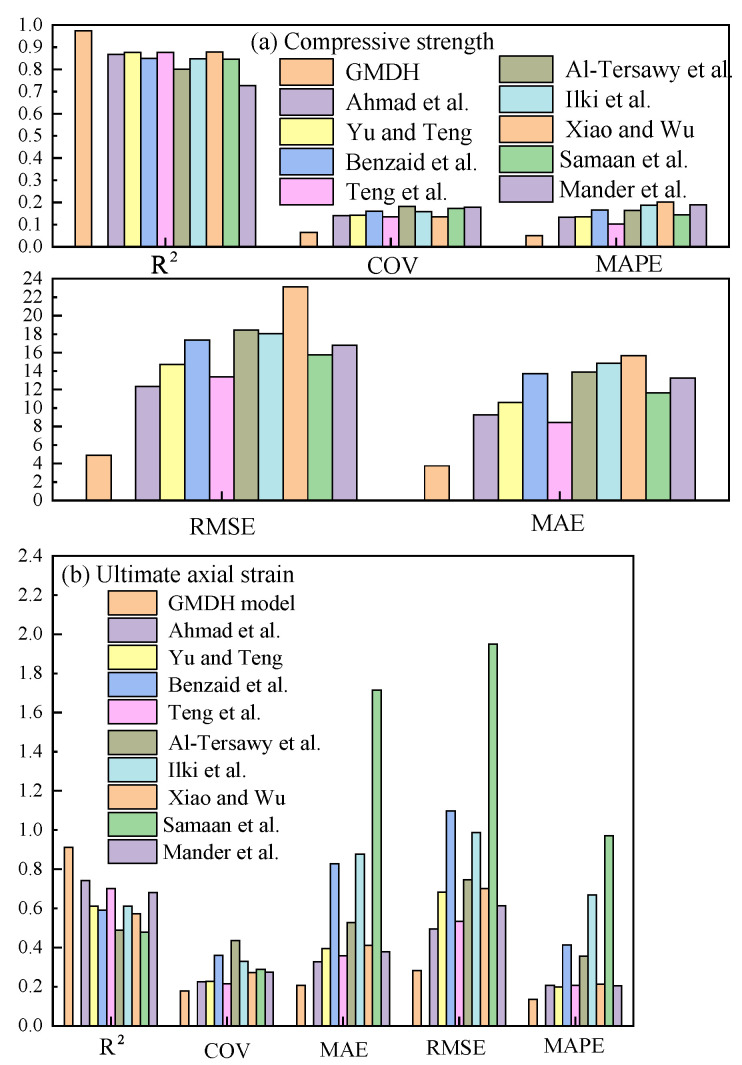
Comparison results of different models under different indexes [4,6,9,10,11,31,52,53,54].

To further evaluate whether the GMDH model has application potential, it was compared with nine existing confinement models [4,6,9,10,11,31,52,53,54]. The prediction results of the nine confinement models are shown in Figure 10, and the comparison results with the GMDH model are shown in Figure 11.

It can be seen from Figure 10 and Figure 11 that the GMDH model has great application potential in the prediction of ultimate conditions. The prediction results of the GMDH model are better than those of the nine confinement models, especially in the ultimate axial strain prediction, showing great superiority. The nine existing confinement models perform well in the compressive strength prediction, and the prediction results agree well with the measured results. However, in the ultimate axial strain prediction, the prediction results of the nine existing models are very poor, and the determination coefficients are all below 0.75. The good performance of the GMDH model in the ultimate axial strain prediction can provide a more accurate reference for engineering design.

### 5.6. Strain Reduction Coefficient Model

Among the eleven input parameters of the GMDH model, it is not easy to obtain the hoop strain of the FRP jacket at rapture $\varepsilon_{h,rup}$. Therefore, the strain reduction coefficient $K_{\varepsilon ,f}$ is introduced to estimate the $\varepsilon_{h,rup}$ according to the fracture strain $\varepsilon_{FRP}$ of the material property test, and the calculation formula is as follows:

(13) $$\varepsilon_{h,rup}=\frac{\varepsilon_{FRP}}{K_{\varepsilon ,f}}$$

The determination of the strain reduction coefficient has been paid much attention by many scholars [56,57,58,59,60,61,62,63]. Sadeghian et al. [58] proposed a strain reduction coefficient of 0.70 to replace the 0.55 in ACI 440.2R-17 through experimental research and statistical analysis. Sadeghian et al. [59] also proposed a simplified strain reduction coefficient model with the diameter of the concrete column and the total thickness of the FRP jacket as the independent variables. In addition, Lim and Ozbakkaloglu [60], Li et al. [61], Zhou et al. [62] and Yuan et al. [63] proposed four strain reduction coefficient prediction formulas, as shown in Table 4.

**Figure 12:**
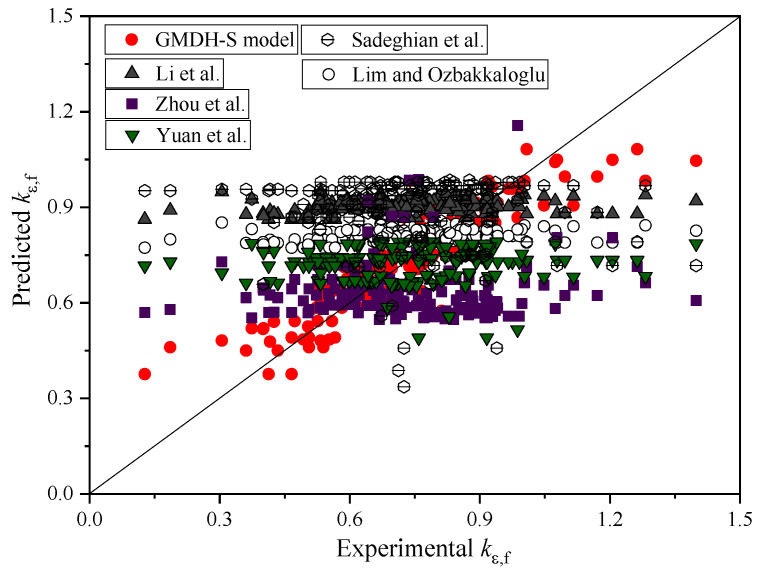
Prediction results of different strain reduction coefficient models [59,60,61,62,63].

Referring to the selection of the input parameters of the models proposed by Zhou et al. [62], this paper took four parameters (i.e., concrete compressive strength $f_{co}^{\prime }$, the elastic modulus of the FRP jacket $E_{FRP}$, the diameter of the concrete core D and the total thickness of the FRP jacket $T_{FRP}$) as the input parameters, and established a new strain reduction coefficient model through GMDH (GMDH-S model). The prediction results of the GMDH-S model and five existing prediction formulas are shown in Table 5 and Figure 12. It can be seen that the prediction accuracy of five existing prediction models is low, and the R^2^, COV, MAE, RMSE and MAPE values of the GMDH-S model are all better compared to those of the five existing models.

### 5.7. Graphical User Interface (GUI)

**Figure 13:**
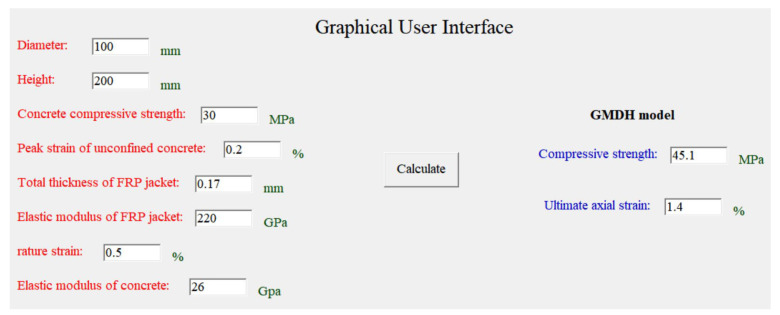
Graphical user interface (GUI).

For practical applications, a graphical user interface (GUI) (as shown in Figure 13) was developed. Since it is freely available, interested users can download the software for practical use. The use of the application is simple. First, the values of the eight parameters are entered. Then, the button “Calculate” is clicked, and the prediction values of the compressive strength and ultimate axial strain will be displayed in the corresponding position. Considering that some parameters such as the confining stress, stiffness ratio and strain ratio are related to other original parameters, only the original parameters need to be input into the GUI.

## 6. Conclusions

This paper established a database containing the experimental data of 221 FRP-confined normal concrete cylinder specimens from the available literature. Based on this database, the performances of nine existing confinement models from the published literature were discussed, and five comprehensive indicators were selected to evaluate these models. In addition, this paper applied a promising machine learning technique (i.e., GMDH) to establish a novel confinement model. Several notable findings of this paper are as follows:

(1) The input form has a great influence on the prediction results of the model. Therefore, the influence of the input form needs to be considered when establishing the confinement model by machine learning techniques.
(2) The nine existing confinement models perform well with regards to the prediction of compressive strength and can provide an effective reference for practical applications. However, they are slightly insufficient in the prediction of ultimate axial strain, and the determination coefficients of all the models are lower than 0.75.
(3) The prediction results of the GMDH model agree well with the experimental results, and the R^2^, COV, MAE, RMSE and MAPE values of the GMDH model are 0.974, 0.065, 3.742, 4.904 and 0.051 (compressive strength), and 0.911, 0.177, 0.208, 0.283 and 0.135 (ultimate axial strain), respectively, which are better compared to the existing confinement models.
(4) The strain reduction coefficient model established based on GMDH is better than the existing literature models, and the R^2^, COV, MAE, RMSE and MAPE values are 0.817, 0.134, 0.066, 0.089 and 0.095, respectively.
(5) The GMDH-type neural network has good application potential regarding the prediction of the ultimate conditions of FRP-confined concrete. However, a too complicated formula may affect the promotion of the GMDH model. Therefore, for practical applications, this paper developed a graphical user interface (GUI) based on Visual Basic 6.0 software, which is freely available.
(6) Similar to other models based on machine learning algorithms, the GMDH model also has some limitations. The application scope of the model is limited to FRP-confined normal-strength concrete, and the values of each input parameter should be within the range of the database.

## Figures and Tables

**Table 1:** Results of the Pearson correlation analysis.

Parameters	$f_{cc}^{\prime }$	$\varepsilon_{cu}$	Parameters	$f_{cc}^{\prime }$	$\varepsilon_{cu}$
*D*	0.112	−0.121	$\varepsilon_{h,rup}$	0.006	0.377 ^#^
*H*	−0.104	−0.127	$f_{l}$	0.928 ^#^	0.653 ^#^
$f_{co}^{\prime }$	0.320 ^#^	−0.029	$\rho_{k}$	0.598 ^#^	0.378 ^#^
$\varepsilon_{co}$	0.012	0.237 ^#^	$\rho_{\varepsilon }$	0.023	0.301 ^#^
$E_{c}$	0.303 ^#^	−0.003	$f_{cc}^{\prime }$	1	0.676 ^#^
$T_{FRP}$	0.563 ^#^	0.305 ^#^	$\varepsilon_{cu}$	0.676 ^#^	1
$E_{FRP}$	−0.101	−0.169 *			

Note: * and ^#^ indicate that the correlation is significant at the 0.05 and 0.01 levels (two-tailed), respectively.

**Table 2:** Existing confinement models.

Model and Year	Compressive Strength	Ultimate Axial Strain
Ahmad et al. [10] 2020	$f_{cc}^{\prime }=f_{co}^{\prime }+3.1f_{co}^{\prime }{\left(\frac{f_{l}}{f_{co}^{\prime }}\right)}^{0.83}$	$\varepsilon_{cu}=\left(1.85+7.46\rho_{\varepsilon }^{1.171}\rho_{k}^{0.71}\right)\varepsilon_{co}$
Yu and Teng [11] 2011	$f_{cc}^{\prime }=f_{co}^{\prime }-\left(22.75\frac{f_{co}^{\prime }}{E_{l}}-3.5\right)E_{l}\varepsilon_{h,rup}$	$\varepsilon_{cu}=0.6{\left(\frac{E_{l}}{f_{co}^{\prime }}\right)}^{0.8}{\left(\varepsilon_{h,rup}\right)}^{1.45}+0.0033$
Benzaid et al. [6] 2010	$f_{cc}^{\prime }=f_{co}^{\prime }+2.2f_{l}$	$\varepsilon_{cu}=2\varepsilon_{co}+7.6\frac{f_{l}}{f_{co}^{\prime }}\varepsilon_{co}$
Teng et al. [9] 2009	$f_{cc}^{\prime }=f_{co}^{\prime }+3.5f_{co}^{\prime }\left(\rho_{k}-0.01\right)\rho_{\varepsilon }$	$\varepsilon_{cu}=1.75\varepsilon_{co}+6.5\varepsilon_{co}\rho_{\varepsilon }^{1.45}\rho_{k}^{0.8}$
Al-Tersawy et al. [52] 2007	$f_{cc}^{\prime }=f_{co}^{\prime }+1.96f_{co}^{\prime }{\left(\frac{f_{l}}{f_{co}^{\prime }}\right)}^{0.81}$	$\varepsilon_{cu}=2\varepsilon_{co}+8.16{\left(\frac{f_{l}}{f_{co}^{\prime }}\right)}^{0.34}\varepsilon_{co}$
Ilki et al. [53] 2004	$f_{cc}^{\prime }=f_{co}^{\prime }+2.4f_{co}^{\prime }{\left(\frac{f_{l}}{f_{co}^{\prime }}\right)}^{1.2}$	$\varepsilon_{cu}=\varepsilon_{co}+20{\left(\frac{f_{l}}{f_{co}^{\prime }}\right)}^{0.5}\varepsilon_{co}$
Xiao and Wu [31] 2000	$f_{cc}^{\prime }=1.1f_{co}^{\prime }+\left(4.1-0.75\frac{f_{co}^{\prime }}{E_{l}}\right)f_{l}$	$\varepsilon_{cu}=\frac{\varepsilon_{h,rup}-0.0005}{7{\left(\frac{f_{co}^{\prime }}{E_{l}}\right)}^{0.8}}$
Samaan et al. [54] 1998	$f_{cc}^{\prime }=f_{co}^{\prime }+6f_{l}^{0.70}$	$\varepsilon_{cu}=\frac{f_{cc}^{\prime }-0.872f_{co}^{\prime }-0.371f_{l}-6.258}{245.61f_{co}^{\prime }{}^{0.2}+1.3456\frac{E_{FRP}T_{FRP}}{D}}$
Mander et al. [4] 1988	$f_{cc}^{\prime }=\left[2.254{\left(1+7.94\frac{f_{l}}{f_{co}^{\prime }}\right)}^{0.5}-2\frac{f_{l}}{f_{co}^{\prime }}-1.254\right]f_{co}^{\prime }\ldots \varepsilon_{cu}=\left(5\frac{f_{cc}^{\prime }}{f_{co}^{\prime }}-4\right)\varepsilon_{co}$	

**Table 3:** Input forms.

Model	$\mathrm{Compressive}\;\mathrm{Strength}\;f_{cc}^{\prime }$	$\mathrm{Ultimate}\;\mathrm{Axial}\;\mathrm{Strain}\;\varepsilon_{cu}$
A	$D,H,f_{co}^{\prime },\varepsilon_{co},E_{c},T_{FRP},E_{FRP},\varepsilon_{h,rup}$	
B	$f_{l},\rho_{k},\rho_{\varepsilon }$	
C	$f_{co}^{\prime },\;\rho_{k},\rho_{\varepsilon }$	$\varepsilon_{co},\;\rho_{k},\rho_{\varepsilon }$
D	$D,H,f_{co}^{\prime },\varepsilon_{co},E_{c},T_{FRP},E_{FRP},\varepsilon_{h,rup},f_{l},\rho_{k},\rho_{\varepsilon }$	

**Table 4:** Existing strain reduction coefficient models.

Model	Year	Equation
Sadeghian et al. [59]	2018	Kε,f=[1+20(TFRPD)]−1
Lim and Ozbakkaloglu [60]	2014	$K_{\varepsilon ,f}=0.9-2.3f_{co}^{\prime }\times 10^{-3}-0.75E_{FRP}\times 10^{-6}$
Li et al. [61]	2016	$K_{\varepsilon ,f}=1-0.0025f_{co}^{\prime }$
Zhou et al. [62]	2019	$K_{\varepsilon ,f}=0.5322+0.0039\rho$
Yuan et al. [63]	2021	$K_{\varepsilon ,f}=0.83-1.14f_{co}^{\prime }\times 10^{-3}-486.4E_{FRP}\times 10^{-6}$

**Table 5:** Comparison of the accuracy of strain reduction coefficient under different metrics.

Model	R^2^	COV	MAE	RMSE	MAPE
GMDH-S model	0.817	0.134	0.066	0.089	0.095
Sadeghian et al. [59]	0.007	0.526	0.227	0.277	0.413
Lim and Ozbakkaloglu [60]	0.046	0.457	0.165	0.208	0.302
Li et al. [61]	0.049	0.457	0.207	0.255	0.387
Zhou et al. [62]	0.002	0.452	0.191	0.237	0.275
Yuan et al. [63]	0.002	0.476	0.159	0.204	0.266

## Data Availability

The raw/processed data required to reproduce these findings will be made available on request.

## References

[1] Hasan H.A., Sheikh M.N., Hadi M.N.S. (2019). Maximum axial load carrying capacity of fiber reinforced-polymer (FRP) bar reinforced concrete columns under axial compression. Structures.

[2] Zhou Y., Liu X., Xing F., Cui H., Sui L. (2016). Axial compressive behavior of FRP-confined lightweight aggregate concrete: An experimental study and stress-strain relation model. Constr. Build. Mater..

[3] Chen G.M., He Y.H., Jiang T., Lin C.J. (2016). Behavior of CFRP-confined recycled aggregate concrete under axial compression. Constr. Build. Mater..

[4] Mander J.B., Priestley M.J.N., Park R. (1988). Theoretical stress-strain model for confined concrete. J. Struct. Eng..

[5] Guler S. (2014). Axial behavior of FRP-wrapped circular ultra-high performance concrete specimens. Struct. Eng. Mech..

[6] Benzaid R., Mesbah H., Chikh N.E. (2010). FRP-confined concrete cylinders: Axial compression experiments and strength model. J. Reinf. Plast. Compos..

[7] Naderpour N., Nagai K., Fakharian P., Haji M. (2019). Innovative models for prediction of compressive strength of FRP-confined circular reinforced concrete columns using soft computing methods. Compos. Struct..

[8] Zhao J.L., Yu T., Teng J.G. (2014). Stress-strain behavior of FRP-confined recycled aggregate concrete. J. Compos. Constr..

[9] Teng J.G., Jiang T., Lam L., Luo Y.Z. (2009). Refinement of a design-oriented stress-strain model for FRP-confined concrete. J. Compos. Constr..

[10] Ahmad A., Plevris V., Khan Q.U.Z. (2020). Prediction of properties of FRP-confined concrete cylinders based on artificial neural networks. Crystals.

[11] Yu T., Teng J.G. (2010). Design of concrete-filled FRP tubular columns: Provisions in the Chinese technical code for infrastructure application of FRP composites. J. Compos. Constr..

[12] Fan X.L., Wu Z.M., Wu Y.F., Zheng J.J. (2013). FRP-confined Concrete Cylinders: Axial Compression Experiments and Strength Model. Comput. Concr..

[13] Spoelstra M.R., Monti G. (1999). FRP-confined concrete model. J. Compos. Constr..

[14] Realfonzo R., Napoli A. (2011). Concrete confined by FRP systems: Confinement efficiency and design strength models. Compos. Plan B Eng..

[15] Khorramian K., Sadeghian P. (2021). New mechanics-based confinement model and stress–strain relationship for analysis and design of concrete columns wrapped with FRP composites. Structures.

[16] Cardellicchio A., Ruggieri S., Leggieri V., Uva G. (2022). View VULMA: Data set for training a machine-learning tool for a fast vulnerability analysis of existing buildings. Data.

[17] Ruggieri S., Cardellicchio A., Leggieri V., Uva G. (2021). Machine-learning based vulnerability analysis of existing buildings. Autom. Constr..

[18] Sun H., Burton H.V., Huang H. (2020). Machine learning applications for building structural design and performance assessment: State-of-the-art review. J. Build. Eng..

[19] Keshtegar B., Sadeghian P., Gholampour A., Ozbakkaloglu T. (2017). Nonlinear modeling of ultimate strength and strain of FRP-confined concrete using chaos control method. Compos. Struct..

[20] Jawdhari A., Hadhood A., Fam A. (2021). Confinement model for FRP-wrapped circular columns when the wraps are subjected to damage. Constr. Build. Mater..

[21] Sadeghian P., Fam A. (2015). Improved design-oriented confinement models for FRP-wrapped concrete cylinders based on statistical analyses. Eng. Struct..

[22] Mansouri I., Ozbakkaloglu T., Kisi O., Xie T. (2016). Predicting behavior of FRP-confined concrete using neuro fuzzy, neural network, multivariate adaptive regression splines and M5 model tree techniques. Mater. Struct..

[23] Cevik A., Cabalar A.F. (2008). A genetic-programming-based formulation for the strength enhancement of fiber-reinforced-polymer-confined concrete cylinders. J. Appl. Polym. Sci..

[24] Cevik A., Göğüş M.T., Güzelbey İ.H., Filiz H. (2010). Soft computing based formulation for strength enhancement of CFRP confined concrete cylinders. Adv. Eng. Softw..

[25] Gandomi A.H., Alavi A.H., Sahab M.G. (2010). New formulation for compressive strength of CFRP confined concrete cylinders using linear genetic programming. Mater. Struct..

[26] Jiang T., Teng J.G. (2007). Analysis-oriented models for FRP confined concrete: A comparative assessment. Eng. Struct..

[27] Lam L., Teng J.G. (2004). Ultimate condition of fiber reinforced polymer-confined concrete. J. Compos. Constr..

[28] Lam L., Teng J.G., Cheung C.H., Xiao Y. (2006). FRP-confined concrete under axial cyclic compression. Cem. Concr. Compos..

[29] Teng J.G., Yu T., Wong Y.L., Dong S.L. (2007). Hybrid FRP-concrete–steel tubular columns: Concept and behaviour. Constr. Build. Mater..

[30] Saadatmanesh H., Ehsani M.R., Li M.W. (1994). Strength and ductility of concrete columns externally reinforced with fiber composite straps. ACI Struct. J..

[31] Xiao Y., Wu H. (2000). Compressive behavior of concrete confined by carbon fiber composite jackets. J. Mater. Civ. Eng..

[32] Picher F., Rochette P., Labossiere P. (1996). Confinement of concrete cylinders with CFRP. Proceedings of the First International Conference on Composites for Infrastructures.

[33] Watanable K., Nakamura H., Honda T., Toyoshima M., Iso M., Fujimaki T. Confinement effect of FRP sheet on strength and ductility of concrete cylinders under uniaxial compression. Proceedings of the Third International Symposium on Non-Metallic (FRP) Reinforcement for Concrete Structures.

[34] Matthys S., Taerwe L., Audenaert K. (1999). Tests on axially loaded concrete columns confined by fiber reinforced polymer sheet wrapping. Proceedings of the Fourth International Symposium on Fiber Reinforced Polymer Reinforcement for Reinforced Concrete Structures.

[35] Rochette P., Labossiere P. (2000). Axial testing of rectangular column models confined with composites. J. Compos. Constr..

[36] Aire C., Gettu R., Casas J.R. (2001). Study of the compressive behavior of concrete confined by fiber reinforced composites. Proceedings of the International Conference on Composites in Construction.

[37] Dias D.S.V., Santos J.M.C. (2001). Strengthening of axially loaded concrete cylinders by surface composites. Proceedings of the International Conference on Composites in Construction.

[38] Micelli F., Myers J.J., Murthy S. (2001). Effect of environmental cycles on concrete cylinders confined with FRP. Proceedings of the International Conference on Composites in Construction.

[39] Pessiki S., Harries K.A., Kestner J.T., Sause R., Ricles J.M. (2001). Axial behavior of reinforced concrete columns confined with FRP jackets. J. Compos. Constr..

[40] Wang P., Cheong K.K. (2001). RC columns strengthened by FRP under uniaxial compression. Proceedings of the International Conference on FRP Composites in Civil Engineering.

[41] Lorenzis D.L., Micelli F., La T.A. (2002). Influence of specimen size and resin type on the behavior of FRP-confined concrete cylinders. Proceedings of the First International Conference on Advanced Polymer Composites for Structural Applications in Construction.

[42] Shehata I.A.E.M., Carneiro L.A.V., Shehata L.C.D. (2002). Strength of short concrete columns confined with CFRP sheets. Mater. Struct..

[43] Wang W., Wu C., Liu Z., Si H. (2018). Compressive behavior of ultra-high performance fiber reinforced concrete (UHPFRC) confined with FRP. Compos. Struct..

[44] Cui C., Sheikh S.A. (2010). Experimental study of normal-and high-strength concrete confined with fiber-reinforced polymers. J. Compos. Constr..

[45] Ozbakkaloglu T., Lim J.C. (2013). Axial compressive behavior of FRP-confined concrete: Experimental test database and a new design-oriented model. Compos. Part B Eng..

[46] Ivakhnenko A.G., Ivaknenko G.A. (1995). The review of problems solvable by algorithms of the group method of data handling (GMDH). Pattern Recognit..

[47] Hossein R., Rahmat M., Hassan A. (2021). Point-load test and UPV for compressive strength prediction of recycled coarse aggregate concrete via generalized GMDH-class neural network. Constr. Build. Mater..

[48] Rahmat M., John H.B., Reza G. (2012). Prediction of the concrete compressive strength by means of core testing using GMDH-type neural network and ANFIS models. Comput. Mater. Sci..

[49] Farlow S.J. (1984). Self-Organizing Method in Modelling: GMDH Type Algorithms.

[50] American Concrete Institute (ACI) (2008). Guide for the Design and Construction of Externally Bonded FRP Systems for Strengthening Concrete Structures.

[51] Popovics S. (1973). Numerical approach to the complete stress-strain relation for concrete. Cem. Concr. Res..

[52] Al-Tersawy S.H., Hodhod O.A., Hefn-Awy A.A. (2007). Reliability and code calibration of RC short columns confined with CFRP wraps. Proceedings of the 8th International Symposium on Fiber Reinforced Polymer Reinforcement for Reinforced Concrete Structures.

[53] Ilki A., Kumbasar N., Koc V. (2004). Low strength concrete members externally confined with FRP sheets. Struct. Eng. Mech..

[54] Samaan M., Mirmiran A., Shahawy M. (1998). Model of concrete confined by fiber compo-sites. J. Struct. Eng..

[55] Ma G., Liu K. (2021). Prediction of compressive strength of CFRP-confined concrete columns based on BP neural network. J. Hunan Univ..

[56] Smith S.T., Kim S.J., Zhang H.W. (2010). Behavior and effectiveness of FRP wrap in the confinement of large concrete cylinders. J. Compos. Constr..

[57] Chen J.F., Li S.Q., Bisby L.A., Ai J. (2011). FRP rupture strains in the split-disk test. Compos. Part B Eng..

[58] Sadeghian P., Fillmore B. (2018). Strain distribution of basalt FRP-wrapped concrete cylinders. Case Stud. Constr. Mater..

[59] Sadeghian P., Seracino R., Das B., Lucier G. (2018). Influence of geometry and fiber properties on rupture strain of cylindrical FRP jackets under internal ICE pressure. Compos. Struct..

[60] Lim J.C., Ozbakkaloglu T. (2014). Confinement model for FRP-confined high-strength concrete. J. Compos. Constr..

[61] Li P., Wu Y.F., Gravina R. (2016). Cyclic response of FRP-confined concrete with post-peak strain softening behavior. Constr. Build. Mater..

[62] Zhou Y., Zheng Y., Sui L. (2019). Behavior and modeling of FRP-confined ultra-light weight cement composites under monotonic axial compression. Compos. Part B Eng..

[63] Yuan W., Han Q., Bai Y., Du X., Liu Q.L. (2022). A unified confinement model of FRP-wrapped concrete cylinder. China J. Highw. Transp..

